# Embedding Pharmacogenetics Into Clinical Practice to Improve Patient Outcomes

**DOI:** 10.1111/ahg.12601

**Published:** 2025-05-13

**Authors:** John Henry McDermott, Videha Sharma, Jessica Keen, William Gerard Newman

**Affiliations:** ^1^ Division of Evolution, Infection and Genomics, School of Biological Sciences University of Manchester Manchester UK; ^2^ Manchester Centre for Genomic Medicine St Mary's Hospital, Manchester University NHS Foundation Trust Manchester UK; ^3^ Centre for Health Informatics, Division of Informatics, Imaging and Data Science University of Manchester Manchester UK

**Keywords:** adverse drug reaction, adoption, drug optimisation, implementation, effectiveness, pharmacogenomics

## Abstract

Pharmacogenomics, the use of germline genomic data to guide prescription to improve effective and safer medication, holds promise as a clinical intervention. To date in most health systems, there has been limited uptake of pharmacogenomic testing confined to a few single drug–gene associations. Here, we describe the current reactive model of single gene testing and the potential for this to change to a pre‐emptive panel or genome‐based approach. For this change to occur, three major challenges need to be addressed—the pharmacogenomic testing approach, the digital and data integration, and service delivery models. We explore some of the potential solutions and how pharmacogenomics can be integrated into routine care at scale for patient benefit.

## 1 Introduction

Making a diagnosis of a rare genetic condition can sometimes result in information that guides the safe prescription of certain medications (Newman et al. 2012). For example, identifying that an individual has a prolonged QT interval on their ECG will indicate to the clinician that up to 400 medications should be avoided or used with caution to prevent further QT prolongation and the risk of a fatal arrhythmia (2). The number of inherited conditions in which such prescribing information is relevant is small (Table 1) and each of these conditions is rare at a population level. Methods to ensure that this information is communicated to patients and their health professionals have been developed, including physical tools such as medical alert bracelets and information leaflets. However, it is difficult to estimate the reach and effectiveness of these. In the context of the digital age of healthcare, there remains a lack of comprehensive technological solutions, such as alerts embedded within electronic health records that could support safe prescribing for all individuals affected by these rare conditions.

**TABLE 1:** Examples of rare inherited conditions where the diagnosis can guide information about safe prescribing.

Inherited condition	Drug(s) to avoid and/or use with caution	Side effect	Reference
Long QT syndrome (OMIM 192500)	Many including antiarrhythmics e.g. sotalol, flecainide, amiodarone; antibiotics e.g. erythromycin, ciprofloxacin; antidepressants e.g. sertraline, fluoxetine; chemotherapy e.g. tamxifen; anti‐histamines e.g. terfenadine; stimulants e.g. amphetamine; for comprehensive list see https://crediblemeds.org	QT prolongation resulting in torsades de pointes	Woosley et al. 2025
Acute porphyria (OMIM 176000)	Many including tricyclic antidepressants e.g. amitriptyline, barbiturates e.g. thiopental, anabolic steroids, sulphonamides e.g. co‐trimoxazole, taxanes e.g. paclitaxel See British National formulary, expert porphyria resource for comprehensive list	Acute attack resulting in pain, vomiting, hypertension, confusion	McColl and Moore (1981)
Central core disease (OMIM 117000) and multiminicore disease (OMIM 255320	volatile anaesthetics e.g. halothane, isoflurane, either alone or in conjunction with a depolarizing muscle relaxant (specifically, succinylcholine)	Hyperthermia, muscle rigidity, tachycardia can result in rhabdomyolysis and renal failure	Rosenberg et al. 2020
Glucose 6 phosphate dehydrogenase (G6PD) deficiency (OMIM 305900)	Many including antibiotics e.g. chloramphenicol, antimalarials e.g. primaquine, and nonsteroidal anti‐inflammatory drugs e.g. aspirin and ibuprofen for comprehensive list see https://www.g6pd.org	Anaemia, jaundice	Luzzatto and Seneca (2014)
Gilbert syndrome (OMIM 143500)	irinotecan	Increased toxicity—diarrhoea and neutropenia	Strassburg 2008
Marfan syndrome (OMIM 154700) and other familial thoracic aortic aneurysm disorders	fluoroquinolone antibiotics e.g. ciprofloxacin	Increased risk of aortic dissection	Lee et al. 2018
Cockayne syndrome (OMIM 216400) and xeroderma pigmentosum (OMIM 278700)	metronidazole	Acute liver failure	Wilson et al. 2015; Abiona et al. 2021

In contrast to specific inherited conditions where genetic information or a robust clinical diagnosis might guide prescription, there are several genes that are not associated with a clinical phenotype but can predispose to adverse drug reactions or reduced effectiveness of medicines. This concept of pharmacogenetics has been formally recognised since the 1950s (Motulsky 1964) and indeed it was the exposure of African‐American soldiers to primaquine resulting in haemolytic anaemia which highlighted this as clinically relevant (Motulsky 1972).

The discovery that the hepatic cytochrome P450 enzymes were key to the first‐pass metabolism of many commonly prescribed medicines further drove the field forward. However, enzyme testing was not technically feasible on clinically accessible samples such as blood, and progress faltered. Advances in genomic technologies, with access to cheap, rapid and scalable genotyping, revealed the diverse genetic variation of the CYP450 genes and their correlation with enzymatic function (Nebert and Russell 2002). Associations between variants in the CYP450 and other genes coding for pharmacokinetic enzymes, and response to many medications have been reported (Tornio and Backman 2018) and replicated across different populations. However, the adoption of this knowledge into clinical practice has been slow and piecemeal.

## 2 Pharmacogenetics in Current Clinical Practice

In the UK testing for a small number of gene–drug pairs has been introduced into clinical practice. Initially, such testing was undertaken in a retrospective manner—a patient experienced a severe adverse reaction, the clinician noted this and sought an explanation by requesting a genetic test, often undertaken in a research laboratory. Of course, such testing may provide an explanation but does nothing to prevent the initial event which may have resulted in severe morbidity or even death. By contrast, moving to a preventative model where testing is undertaken prior to drug prescription has advantages. To facilitate this, test information must be available at the point of prescription. The test results need to be generated in a clinically relevant timeframe and be easily understandable to the clinician and linked directly to prescribing practices.

To date, in most health services across the world where pharmacogenetic data are available, these are generated as part of a reactive model of service delivery, such that the request for the test is undertaken at the point when a prescription is considered. Hence, a window of opportunity must be available such that the drug is not required immediately but in a time frame within which the result is available. A typical example has been the use of pharmacogenetics to guide thiopurine drug prescription i.e. azathioprine and 6‐mercaptopurine.

In the 1980s absent enzyme activity of thiopurine methyltransferase was shown to correlate directly with severe neutropenia in individuals exposed to standard doses of thiopurines (Weinshilboum and Sladek 1980). One in 300 individuals has this deficiency, usually due to a combination of relatively common variants in TPMT (Yates et al. 1997). Most individuals who are started on thiopurine treatment are using the medication as an immunosuppressant in the context of inflammatory disease and are being weaned off steroid medication. Therefore, there is a window of a few days for the test to be requested and the result made available before a prescription is required. Despite the compelling evidence of a robust drug–gene association with this severe adverse reaction, in the UK it took ∼30 years for broad clinical adoption.

Barriers to the adoption of either genetic or enzyme‐based TPMT testing included the availability and cost of the test, the turnaround time of testing and knowledge among health professionals (Newman and Payne 2008). Professional guidance from gastroenterologists, rheumatologists and dermatologists all resulted in a sudden uplift in testing, coincident with increased availability of testing and a reduction in the cost per test. Standardised prescribing guidance for many drug–gene pairs has been created that ensures consistency and draws upon the most robust study evidence. The most widely used guidance has been generated by the Clinical Pharmacogenetics Implementation Consortium (Caudle et al. 2014) and the Dutch Pharmacogenetics Working Group (Swen et al. 2008). Many other countries are considering national versions based on their own drug formularies and local regulatory practices. These are valuable resources but are explicitly guidance‐based on where pharmacogenetic data are already available and do not define the populations to test or how or where this should happen in the clinical pathway. Continued refinement of these guidance documents based on new data, international collaboration and work alongside key stakeholders, including regulatory bodies will be key.

The gestation of TPMT testing adoption contrasts sharply with the international adoption of testing for HLA‐B*5701 prior to abacavir prescription. Evidence of a severe hypersensitivity reaction secondary to abacavir treatment in the context of HIV/AIDS emerged in the early 2000s. Mallal et al. 2002 demonstrated an association with the HLA‐B*5701 allele and a subsequent randomised controlled trial PREDICT‐1 (Mallal et al. 2008), which resulted in regulatory guidance from the FDA, EMA and MHRA that mandated testing prior to prescription. Almost more than any other factor, regulatory guidance drives rapid adoption and leads to changes in clinical practice.

As the use of HLA genotyping increased to guide abacavir treatment, this led to heightened interest as to the value and potential of pharmacogenetics to improve clinical outcomes more broadly. Over the past two decades, many excellent studies defined drug–gene associations, especially rare skin hypersensitivity reactions to carbamazepine (Chung et al. 2004; McCormack et al. 2011) and allopurinol (Hung et al. 2005), drug‐induced liver injury due to flucloxacillin (Daly et al. 2009) and improved outcomes secondary to genotype‐guided warfarin (Pirmohamed et al. 2013) and clopidogrel (Shuldiner et al. 2009) prescription. It is this last example of genotype‐guided antiplatelet therapy which is having the most significant impact (van den Broek et al. 2024). Millions of individuals are prescribed antiplatelets each year to reduce the risk of recurrent stroke, and thrombotic events after percutaneous coronary intervention and in the treatment of peripheral vascular disease. Reduced CYP2C19 metabolism due to loss of function variants is common across all populations and such individuals are predicted to respond less to clopidogrel and benefit from an alternative medication.

Subsequent health economic and implementation studies have considered the clinical utility of these individual tests. All of this work resulted in a landscape where there is variable use of single tests (genetic or enzymatic) across different clinical settings where the results are used to guide a single prescribing decision with minimal re‐use of data to inform future prescribing events.

### 2.1 The Future of Pharmacogenetic Testing

We are now at a crossroads where there is a choice to gradually increase pharmacogenetic testing by adding single genes and integrating test results at the point of prescription or to use broader pharmacogenomic data that can inform decisions at multiple time points through an individual's life. Such choices are now possible due to changes in genomic technologies—wider coverage of genetic variation, increased speed of testing and reduced cost, widespread adoption of electronic health records and use of facilitating technologies, such as clinical decision support systems. It is possible to move from a reactive model with testing initiated at the point of prescription for a single gene relevant to a single drug, towards a pre‐emptive approach where a panel genomic test (consisting of multiple relevant variants) or whole genome sequence is performed at a set point. This would allow the resultant data to be made available to prescribers and the individual, informing the safe and effective prescription of multiple medications across a lifetime, including commonly prescribed drugs like statins, anti‐depressants, analgesics, and anti‐platelets. Such an approach is supported by data from large adult populations, including the UK Biobank of ∼500,000 individuals, where over 99.5% of individuals carry an actionable pharmacogenetic variant and ∼25% have already been prescribed a drug with relevant pharmacogenetic informed prescribing guidance (McInnes et al. 2021).

Data supporting the transition to panel‐based pharmacogenomic testing has been generated through a number of large studies (Van Driest et al. 2014; Wang et al. 2022). Most recently, a European multi‐national centre of ∼7000 individuals demonstrated a reduction of ∼30% in adverse drug reactions (Swen et al. 2023).

To move to this new model, three main challenges—the testing approaches, the digital and data integration, and service delivery models—need to be considered, and their potential solutions explored. Of course, it is appropriate to state at this juncture that the economic considerations of who pays (the individual, insurer or public health system) and what the savings and costs to the health systems in different countries will be key factors in adoption (McDermott, Wright, et al. 2022).

### 2.2 Genomic Testing

At present, the most genetic testing is delivered by laboratories with specific expertise in the analysis of DNA. This tends to be undertaken on individuals affected by, or at risk of, rare inherited conditions or in tumour tissue from individuals affected by cancer to determine the most effective treatment (stratified or personalised medicine).

For many genetic tests, the objective is to identify a rare or novel genetic variant which is responsible for a clinical phenotype. This often means considerable analysis of the resultant genotyping/sequencing data by clinical scientists using multiple databases and algorithms to determine the classification of the genetic variant and its relationship with the clinical features. Such testing has by its nature tended to be relatively low throughput, compared to standard biochemistry or haematological tests, and with turnaround times measured in days to months rather than hours.

Although there has been a large increase in the amount of genomic testing delivered in healthcare over the past decade, there are fundamental differences in how this should be applied in the context of pharmacogenomics. If we are to move to a scenario where pharmacogenomic data are to be made available at a population scale—either for all individuals about to start a pharmacogenomics‐relevant medication or indeed the entire population, then the current laboratory capacity and infrastructure will require a complete overhaul.

Direct analysis of DNA from the tested analyte (blood or saliva) without a DNA extraction step will speed up the process and reduce costs. Automation of laboratory processes so that tens of thousands of samples per week can be analysed—analogous to what happened for Covid‐19 testing during the recent pandemic—will be required. Minimisation of data analysis so that variants are only reported where there is definitive prescription guidance is appropriate and necessary. Pharmacogenomic tests will be requested and acted upon by clinicians who do not have the time and may not have the expertise, to interrogate each result to determine what it means. Returning too much data would result in enormous heterogeneity in how pharmacogenetic data is used and may impact uptake (McDermott et al. 2024a). Hence, standardisation and automation of pharmacogenomic test reporting are paramount. When pharmacogenomic data are generated because of an individual having genetic testing for another reason, e.g. to diagnose a health problem or as part of a genomic screen, there is the potential to return this additional or secondary information to the individual. This is entirely appropriate, but to maximise its value it needs to be presented in a way that makes the information meaningful to the individual and their health professional and leads to a clinical action. Ideally, this information should be integrated into the individual's health care record.

Genomic technology is developing rapidly, with costs reducing and the speed of data generation increasing. In the short term, a genotype‐based approach which includes all variants (including copy number variants) relevant to the drugs used within a healthcare setting and representative of the variants found in the ethnic groups tested should be an achievable approach. That said, whole genome sequencing is becoming more accessible and has the advantage that all (or nearly all) sequence variants are captured. This is especially true of new long‐read approaches which have advantages in complex genomic regions relevant to pharmacogenomics, including the *HLA* locus and *CYP2D6*. Bioinformatic challenges remain for genome sequencing to extract the relevant data, and the costs of large data transfer, analysis and storage need to be considered. The genomic testing approach is an important consideration, but one that will be addressed by commercial developments in conjunction with genomic laboratories.

### 2.3 Digital and Data Integration

Traditionally, genetic test reports have been paper‐based. These reports are typically very detailed, including information about the gene(s) tested, the testing approach, any variants identified and some unstructured interpretative text. Most front‐line clinicians, such as general practitioners or pharmacists, are not familiar with such reports. If pharmacogenomics is to be adopted at scale, information needs to be available to clinicians wherever drug prescribing is undertaken—most of this being in primary care. Furthermore, in a fully pre‐emptive model, the healthcare professional being presented with pharmacogenomic data is unlikely to have requested the test initially.

In work that we have undertaken with health professionals (McDermott, Sharma, Newman, et al. 2024), their preferences are for results to be presented as actionable prescribing guidance, becoming visible at the point of prescription within their electronic healthcare record. Recognising that there are multiple providers of electronic healthcare records and linked clinical decision support tools, there is a need to separate the management of pharmacogenetic data from the various clinical systems that visualise actionable prescribing guidance. This ‘vendor‐neutral’ approach ensures data are not locked into single systems and potentially available across providers in a healthcare system (Sharma et al. 2021). The lifetime value of pharmacogenomic data further strengthens this argument and policymakers must heed the risk of losing the potential of genomic testing if data are not accessible in the long term. In the context of new findings, especially if whole genome data is available, independent data management of pharmacogenetic results, separate from EHRs and other proprietary solutions means results can be reinterpreted as evidence evolves, without relying on individual vendors to update their systems. (de Mello et al 2022)

Critical to realising this ‘vendor‐neutral’ approach is the need to structure pharmacogenomic laboratory data using open standards—this allows standardised computer‐readable storage of data and shifts away from the traditional paradigm of unstructured ‘reports’ to structured ‘results’. Such structured data can be integrated with existing clinical systems, such as electronic health records, prescribing software and patient portals (Sharma et al. 2025). In collaboration with the Global Alliance for Genomics and Health, we have started to develop these open standards, with an open data model for a ‘pharmacogenetic test result’ published publicly in the openEHR library (Sharma et al. 2024). This work has been influenced by the HL7 FHIR Genomics Reporting Implementation Guide and developing standards for both storage and messaging of pharmacogenetic results is important to support data exchange across systems, and ensure results follow the patient across care settings.

This ‘interoperable’ data strategy, combined with the user‐centred design of clinical decision support systems that presents pharmacogenomic data at the right time, to the right person will help to bridge the gap from insights to clinical action. An additional benefit of such a strategy is the opportunity to provide patients access to this information through the various digital channels they already use and to engage with their health, including through patient apps and personal health records. Enabling patient access to health data supports understanding and trust, as well as empowers individuals in their healthcare choices (Carini et al. 2021). Finally, to maintain version control of adopted guidelines across a healthcare system, such as the NHS, a centralised informatics solution will enable the guidance to be administered and updated uniformly, minimising the risk of inequity.

### 2.4 Service

For the value of pharmacogenomics to be realised, it needs to be adopted in a way that is equitable, maximises benefit and is integrated into a broader strategy of medicine optimisation where information on diagnosis, adherence, co‐medication, co‐morbidities, height, weight, sex, smoking status, diet are all incorporated to ensure safe effective prescription. Health professionals and the public have both expressed enthusiasm for the adoption of pharmacogenomics into clinical care (McDermott, Sharma, Newman, et al. 2024; McDermott, Sharma, Beaman, et al. 2024). Health professionals prioritise the need for pharmacogenomic data to be integrated into routine clinical pathways and information systems so that they are easily accessible. The public prioritises their own access to the data—either through an app or other resource that they can use or share with health providers as necessary.

Pharmacogenomic testing is context‐dependent. In some clinical situations, genomic data to guide prescription can be generated and used at a future point, whereas if the data are not already available through a pre‐emptive testing approach, then an alternative needs consideration. Such a scenario arises when newborn babies require antibiotics to prevent or treat sepsis. Here, rapid prescribing decisions are required, and a point‐of‐care testing approach has been shown to be successfully employed without disrupting care pathways and reducing the incidence of aminoglycoside‐associated ototoxicity (McDermott, Mahaveer, et al. 2022). The fact that genetic information can be generated from a cheek swab, at the bedside by non‐genetics or laboratory experts, opens the opportunity for such testing to be employed in other acute clinical settings like determining optimum anti‐platelet treatment in acute coronary syndrome or stroke.

For pharmacogenomics to be adopted at scale, there will need to be training and education of the healthcare workforce and ongoing engagement with the public. How this is best achieved is uncertain. In many countries where pharmacogenomics has been enthusiastically used, pharmacists have been the key professional group driving adoption, drawing up guidance, and evaluating the consequences of using this information (Cicali et al. 2022). Understanding the training needs of clinicians in primary care where they are used to prescribing a broad range of common medications compared to those in secondary or tertiary healthcare settings where the prescription is often of a smaller range of more specialised medications is important.

Pharmacogenomics will only realise its potential if prescribers use the guidance information. Previous pragmatic studies of *TPMT* genotyping have shown inconsistent application of prescribing advice (Newman et al. 2011). Hence, the importance of presenting information in an accessible format, as described above, and reinforcing the value of the data through education and training of health professionals or potentially by only allowing prescription of certain drugs when acknowledgement of the prescribing advice has been actioned.

### 2.5 Ongoing Challenges

To date, as in most areas of genomic research, most pharmacogenomic evidence has been generated in populations of white Northern European ancestry. For some genes, the same variants are present across populations, albeit at varying allele frequencies, e.g. in *CYP2C19* ∼30% of White Europeans and ∼60% of South Asians carry at least one loss of function CYP2C19*2 allele (Magavern et al. 2023). Therefore, testing the same variant in these populations provides relevant information and does not result in inequity. However, for many pharmacogenetics, there are rare variants that are only present in certain ethnic groups e.g. *DPYD* (Chan et al. 2024), and therefore restriction of the genetic testing approach to only include those present in a certain ethnic group creates inequity where individuals may experience drug‐associated toxicity which could have been avoided if the appropriate testing strategy was in place and associated guidance followed. The Association of Molecular Pathology have made attempts to address this by defining a minimum set of variant alleles to include in clinical pharmacogenomic assays (Pratt et al. 2024).

Large‐scale studies across different ethnic groups are required to identify and collate rare variants before they can be considered for inclusion in testing panels. However, there arises an additional challenge in that many of these variants have uncertain significance in terms of their function and precise prescribing information is not available. Hence, identification of the variant can be achieved, but then how to use that information in clinical practice becomes the barrier. Collation of data on the functional effects of variants can be achieved through genetic epidemiological studies and the Pharmacogene Variation (PharmVar) Consortium has created a valuable resource (Gaedigk et al. 2021). Many groups are undertaking large‐scale functional studies of variation across genes to understand their pathogenicity and linking these to prescribing guidance is going to be one of the great challenges of the next decade (Rubin et al. 2025).

Many drug–gene associations linking genomic variants to drug response remain unknown. Multiple approaches can be made to adopt these using genome‐wide association studies, genome sequencing approaches linked to robust clinical outcomes and prescribing data. Such studies are challenging but can be done in different settings by pharmaceutical companies as they develop new agents as has recently been seen for *CYP2C9* variation guiding siponimod use in patients with multiple sclerosis (Kane 2023); large post‐marketing studies of real‐world data from biobanks encompassing different geo‐ethnic populations; and initiatives linking adverse drug reporting to whole genome sequencing e.g. the Yellow Card Biobank program between the MHRA and Genomics England. Over the next few years, new drug–gene associations will be reported. It is likely that polygenic risk scores will be generated that inform safer and more effective prescriptions. Both are likely to have specificities relevant to different populations. We need to create an adaptable clinical implementation pathway that can seamlessly incorporate these advances without the need for new testing or reporting strategies.

To date, most pharmacogenomic data have been generated and used in the context of starting a new medicine or changing to a new class of drug. In this context, it is clear how using the information might avoid an adverse drug reaction or ensure that a patient is prescribed a more effective medicine. However, for individuals who are already taking one or more medicines, should pharmacogenomic information be used to change a drug portfolio without compelling evidence that the alternative is better? Undertaking pharmacogenomic testing as part of a structured medicine review is enticing, but further evidence is required to ensure that this is a robust strategy resulting in better outcomes, especially in individuals with polypharmacy. Defining these better outcomes will be important—for the individual, these will be the avoidance of adverse drug reactions with their associated morbidity and rarely mortality, and receiving the most effective medication in a timely manner, and avoiding rounds of trial and error before the optimum regimen is achieved. To the health system, these outcomes may be measured by decreased length of hospital stay, decreased need for hospital admission, more cost‐effective clinical pathways resulting in equivalent or better clinical outcomes, reduced medication changes and less need for healthcare interaction. Defining composites of these outcomes will facilitate health economic analyses and more accurately determine the impact of widespread pharmacogenomic testing.

If population‐based pharmacogenomic testing is to be championed as the best strategy to optimise prescribing at scale, then it should be subject to the same challenges as all screening programs (Turnbull et al. 2024). Does it meet Wilson and Jungner (1968) criteria? If so, large‐scale longitudinal pilot programs should be undertaken and assessed to determine the feasibility, economic consequences, and acceptability of such an approach. Should pharmacogenomic testing be introduced as part of newborn screening to create a lifelong repository of data or in middle age where prescription of a drug linked to a relevant pharmacogenetic variant becomes more likely (McDermott, Burke, et al. 2024), or to certain groups in the population where medication is regularly required? A staged introduction rather than full‐scale population testing seems logistically most feasible, but this still requires considerable planning to maximise benefit.

## 3 Summary

To date, pharmacogenomics has been adopted into clinical practice in a modest manner focussed on a small number of drug–gene associations tested via a reactive model of service delivery at the point of prescription. Even in these few examples, there is robust evidence that integrating pharmacogenomics‐guided prescribing into clinical practice results in improved outcomes. Due to improved genomic technologies, the adoption of informatics into healthcare and a rapidly growing evidence base, there are opportunities to expand this approach so that pharmacogenomic testing becomes ubiquitous. It can be envisaged that testing will be delivered in a pre‐emptive context (perhaps to all babies at birth) and is then integrated into a flexible healthcare system that allows the use of this data at multiple points in a person's life. To achieve this, many evidence gaps need to be addressed by professionals working together from many disciplines, including informatics, regulatory science, health economics, genomics and pharmacy, working with public advocates. Investment in implementation is required, and rigorous ongoing evaluation of different approaches will be needed to ensure that the benefits of pharmacogenomics can be realised.

## Author Contributions

All authors contributed to the writing of this review article.

## Conflicts of Interest

Videha Sharma, William Gerard Newman and John Henry McDermott are co‐founders of an early‐stage health technology start‐up, Fava Health Ltd.

## Data Availability

Data sharing not applicable to this article as no datasets were generated or analysed during the current study.
